# Coagulation in Lymphatic System

**DOI:** 10.3389/fcvm.2021.762648

**Published:** 2021-11-24

**Authors:** Wendi Zhang, Jiang Li, Jiangjiu Liang, Xiumei Qi, Jinghui Tian, Ju Liu

**Affiliations:** ^1^Department of Gerontology, The First Affiliated Hospital of Shandong First Medical University and Shandong Provincial Qianfoshan Hospital, Jinan, China; ^2^Medical Research Center, Shandong Medicine and Health Key Laboratory of Microvascular Medicine, Institute of Microvascular Medicine, The First Affiliated Hospital of Shandong First Medical University and Shandong Provincial Qianfoshan Hospital, Jinan, China; ^3^Graduate School, Shandong First Medical University and Shandong Academy of Medical Sciences, Jinan, China; ^4^Qeeloo Medical College, Shandong University, Jinan, China; ^5^Department of Education, Shandong Provincial Qianfoshan Hospital, The First Hospital Affiliated With Shandong First Medical University, Jinan, China; ^6^School of Public Health and Health Management, Shandong First Medical University and Shandong Academy of Medical Sciences, Taian, China

**Keywords:** coagulation, lymphatic endothelium, lymphatic thrombosis, lymph, lymphedema

## Abstract

The lymphatic system maintains homeostasis of the internal environment between the cells in tissues and the blood circulation. The coagulation state of lymph is determined by conditions of coagulation factors and lymphatic vessels. Internal obliteration, external compression or abnormally increased lymphatic pressure may predispose to localized lymphatic coagulation. In physiological conditions, an imbalance of antithrombin and thrombokinase reduces lymphatic thrombosis. However, the release of factor X by lymphatic endothelium injury may trigger coagulation casacade, causing blockage of lymphatic vessels and lymphedema. Heterogeneity of lymphatic vessels in various tissues may lead to distinct levels and patterns of coagulation in specific lymphatic vessels. The quantitative and qualitative measurement of clotting characteristic reveals longer time for clotting to occur in the lymph than in the blood. Cancer, infections, amyloidosis and lymph node dissection may trigger thrombosis in the lymphatic vessels. In contrast to venous or arterial thrombosis, lymphatic thrombosis has rarely been reported, and its actual prevalence is likely underestimated. In this review, we summarize the mechanisms of coagulation in lymphatic system, and discuss the lymphatic thrombosis-related diseases.

## Introduction

The mammalian lymphatic system is a one-way transport system of draining fluid and proteins from the interstitialspaces to the blood circulation. The lymphatic circulation is composed of lymphatic vessels, lymph nodes, lymphocytes, and associated lymphoid organs (1). This system is responsible for reabsorbing exudate tissue fluid from the vascular system through lymphatic capillaries. The fluid is then transported back to the bloodstream to replenish blood supply (2, 3). Although blood vesselsand lymphatic vessels differ greatly in structure, they work together to maintain essential functions, including fluid and protein balance in tissues, cellular nutrition, and proper immune function (4). In blood vessels, the clotting mechanism is a protective mechanism for preventing excessive blood loss while maintaining blood flow (5). However, in the lymphatic vasculature, coagulation is a pathological phenomenon that is closely related to but quite different from blood clotting. In 1914, the presence of thrombin, fibrinogen, and other coagulation factors in the lymph was confirmed, indicating that lymph also has the ability to coagulate (6), while the mechanism of coagulation in the lymphatic system has not been thoroughly studied. In this review, we summarize the possible etiologies, processes, and associated diseases of lymphatic coagulation.

## Structure and Function of the Lymphatic System

The lymphatic system is a unidirectional transit network consisting of a network of vessels and nodes. The lymphatic system is responsible for the reabsorption of plasma components leaking from capillaries and postcapillaryvenules into extracellular spaces (3). When the blood flows through the capillaries, the plasma proteins and part of the liquid are extruded from the vessels into the interstitial fluid because of hydrostatic pressure and osmotic pressure (7, 8). Most of the blood components are reabsorbed by the postcapillaryvenules, while a small portion is absorbed by the lymphatic capillaries to form the lymph (9).

**Figure 1:**
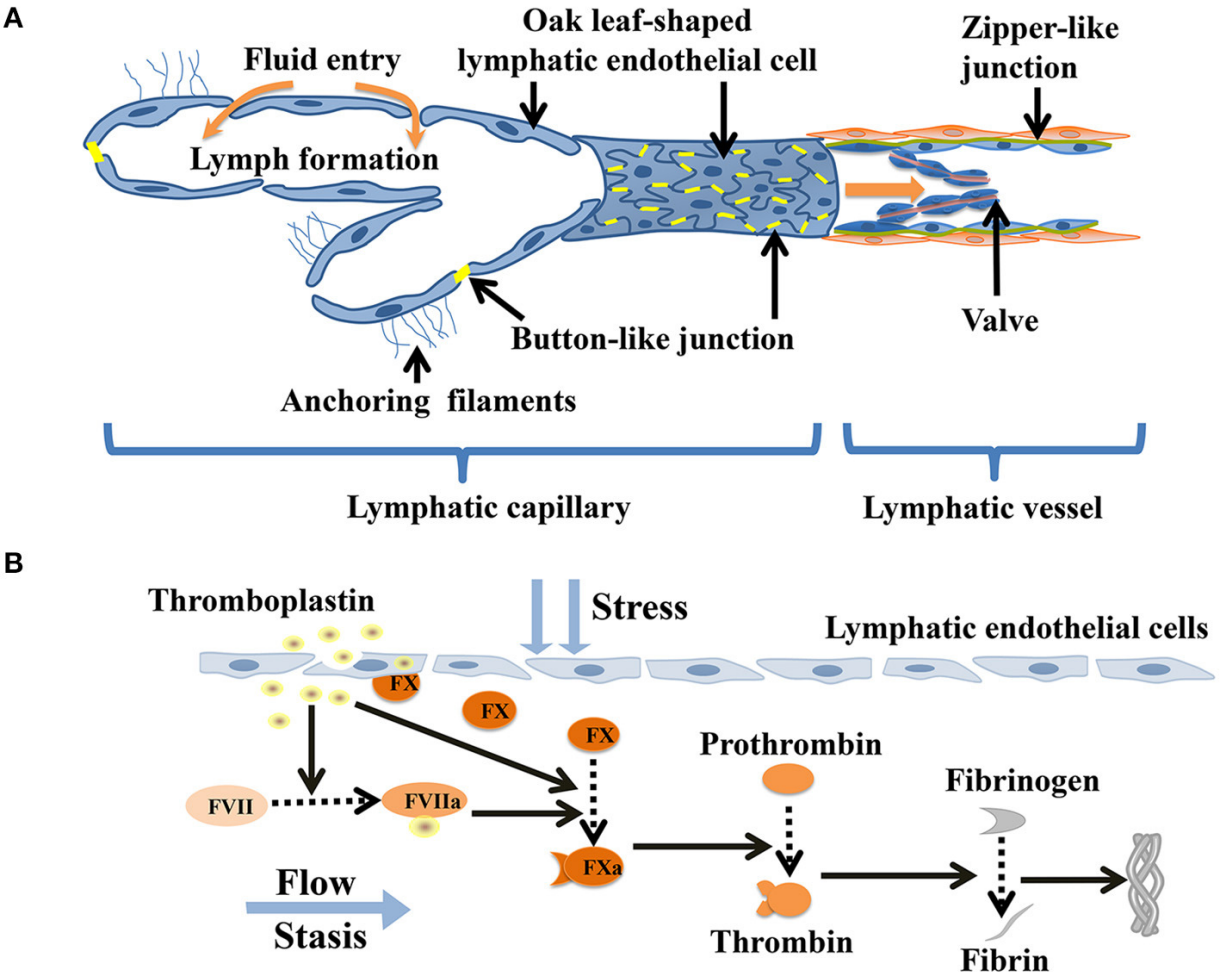
Coagulation in lymphatic vessels. **(A)** Schematic diagram indicating the lymphatic capillaries with oak leaf-shaped endothelial cells and discontinuous button-like junctions. The collecting lymphatic vessels have continuous zipper-like junctions and lymphatic muscle cellscoveragewhich contract and act as intrinsic lymphatic pump, hence facilitating lymph flow. **(B)** Thromboplastin (tissue factor) stimulates damaged lymphatic endothelial cells toreleasefactor X and actives factor VII. Factor X is activated by factor VII-thromboplastin complexes and thromboplastin. Activated factor X helps to convert prothrombin to thrombin, which facilitates theconversion of fibrinogen intofibrin. Eventually, the fibrin mesh forms a dense fibrous protein mass that may cause a large number of embolisms of lymphoid cells.

The lymphatic capillaries are composed of monolayers of discontinuous endothelial cells, which having a relatively flat, “oak leaf” shape (10). The basal membrane is discontinuous and unclear, with no smooth muscle medium, thereby allowing greater permeability (11). The specialized discontinuous buttons junctions serve as anchoring sites at the sides of interdigitated flaps of adjacent oak leaf-shapedendothelial cells (12). The abluminal surface of lymphatic capillaries is notably directly connected to the extracellular matrix by anchoring filaments, which help maintain the shape of the lymphatic vessels and the flow of fluid as the pressure in the matrix changes (11, 13, 14). Pre-collecting lymphatic vessels, located in the deep dermis, drain fluid from the lymphatic capillaries (15). The pre-collecting lymphatics drain into the collecting lymphatic vessels, which are covered with a continuous basement membrane and lymphatic muscle cells. In contrast to capillaries, the collecting lymphatic vessels are lined with lymphatic endothelial cells interconnected by zipper-like junctions between the cell (1, 16, 17). The zipper-like junctions were composed of vascular endothelial cadherin and tight junction–associated proteins. Thesecontinuous zipper-like junctions with basement membrane and intraluminal valves prevent leakage or reflux of lymph during its transport (1, 18). The loosely apposed but overlapping borders of the lymphatic capillaries operate as primary valves that provide unidirectional fluid into lymphatics (19). The smooth muscle layer of collecting lymphatic vessels ensures that the phasic contractions propel lymph forward through the network (20). Action potentials in the lymphatic muscle cells elicit phasic contractions of the collecting lymphatic vessels (11). In addition, cyclical compression and expansion by the surrounding tissue significantly contributes to lymph propulsion, allowing lymph to through collecting pre-nodal lymphatic vessels, lymph nodes and collecting post-nodal lymphatic vessels, to replenish the blood cardiocirculatory system (1) (Figure 1A).

The primary function of the lymphatic system is to replenish the vascular system by regulating tissue fluid balance, facilitating interstitial protein transport, and providing essential immune function (21). To accomplish these functions, lymphatics move fluid and other contents from the interstitium to pass across the nodes and enter the great veins (20). Both extrinsic tissue compression and intrinsic contractions of the lymphatic muscle cells provide the lymphatic system with the energy necessary to overcome the opposing pressure gradients and propel lymph along the lymphatic network (22, 23). When lymphatic vessels are damaged or diseased, edema, fibrosis, immune disorders, nutritional failure, and other conditions may occur (3, 23).

## Blood Coagulation Cascade

The normal blood coagulation process consists of three steps: vascular response, the initiation of platelet plugs, and the formation of fibrin clots (24). Platelet adhesion and aggregation at the site of vascular injury are necessary to stop bleeding (25). When blood vessels are injured, reactive vasoconstriction occurs first, reducing blood flow to the damaged area. Subcutaneous collagen components are also exposed, triggering platelet accumulation and activation (26). Exposureofbloodtotissuefactor (TF) is the major physiological initiatorof blood coagulation. It triggers the production of thrombin, which not only converts fibrinogen to fibrin but also activates platelets (27). Activated platelets secrete aggregatory mediators including thromboxane A2 and adenosine diphosphate. These mediators may provide ligands for platelet adhesion, diffusion, and activation when exposed to circulating blood (28, 29). Platelet adhesion and aggregation at the site of injury result in the formation of a primary “platelet embolism” and the binding of coagulation factors during the clotting cascade leads to the formation of a fibrin mesh. This mesh encapsulates and enhances the thrombus formation, prevents bleeding at the wound site, and promotes healing (30).

Coagulation includes both endogenous and exogenous pathways. Each pathway is initiated by different molecules, and both pathways converge into a common pathway that involves coagulation factors II, V, and X, leading to the formation of fibrin (31). Coagulation reactions start with exposure of TF-bearing cells to blood and continues mainly with activated platelets (32). After vascular endothelial injury, in the presence of calcium, factor IXa binds to factor VIIIa and activates factor X. Factor VIIa comes into contact with the exposed or expressed TF to form vitamin K-dependent enzymecomplexes (28). The factor VIIa-TF complexes can also activate factor X directly or indirectly by activating factor IX, but its efficiency is much lower in the absence of factor VIIa. Activated factor X stimulates a small amount of prothrombin to form thrombin (30). At the initial stage of thrombin production, activation of platelets, factor V, and factor VIII trigger a very small amount of thrombin to cause a burst of thrombin production (32). Experiments have revealed that a large number of prothrombin-activated products were detected at a bleeding time of about 4 minutes (33).

Endothelial cells produce anticoagulants and procoagulant molecules to form structures that rapidly activate platelets and clot blood (34). Von Willebrand factor (vWF) is well-known for its crucial roles in hemostasis; it is synthesized in endothelial cells and stored in Weibel-Palade bodies (WPBs) (35, 36). Platelet adhesion is mediated by vWF, which acts as a bridge between exposed subendothelial collagen and platelet receptors (32). The immobilized vWF attaches platelets to the injured area by binding to the glycoproteinIb-IX complex on the platelet surface (37, 38). This process ultimately leads to a stable platelet adhesion through interaction with the platelet collagen receptors glycoprotein VI and glycoprotein Ia-IIaintegrin (39). Hemostasis is a balancing process of anticoagulant and procoagulant factors. On the one hand, quiescent endothelial cells express thromboregulatory protein (TM), tissue plasminogen activator (tPA), tissue factor pathway inhibitor (TFPI), and heparin sulfate. On the other hand, activated endothelial cells express TF, thrombin receptor, vWF, and plasminogen activator inhibitor-1 (PAI-1) to promote hemostasis (40, 41).

## Formation of Lymphatic Thrombosis

An imbalance consisting of an excessive concentration of antithrombin in the lymph and a low concentration ofthrombokinase (activated coagulation factor X) greatly reduces the possibility of lymphatic thrombosis. The lack of anionic phospholipids on the cell surface and low concentrations of thrombinand TFPI also offset the production of fibrin in the lymph (42–44). Immunoelectron microscopy has illustrated that coagulation factor X is attached to the cell surfaces of lymphocytes under normal conditions (45, 46). Lymphatic thrombosis can be triggered by the lymphatic stream in contact with necrosis cells or by infection of the tissues in the neighborhood of the lymphatic vessels. Under these conditions accompanied by a low lymph flow or a hypercoagulable state, the release of coagulation factor X by the disintegration of the lymphatic endothelium significantly furnishes favorable conditions for thrombosis within the lymphatic vessels (42, 47). When necrotic cells in contact with the lymph stream, thromboplastin (i.e., tissue factor) is released and enters the lymph to activate factor VII (46, 48). When free factor VIIa binds to thromboplastin in the context of lymphatic endothelial cells membrane surface, its proteolytic activity is enhanced substantially (48). The factor VIIa- thromboplastin complexes activate factor X released by the injured lymphatic endothelium. Activated factor X stimulates to production of prothrombinand formations of thrombin, which facilitates the conversion of a large amount of fibrinogen to fibrin. Eventually, this process leads to the formation of a compact mass of fibrin containing lymphoid cells (43, 46, 49, 50) (Figure 1B). The formation of lymphatic thrombosis are supported by the release of thromboplastin substances from the injured lymphatic endothelium and the chronic obstruction of lymph flow in the presence of a hypercoagulable milieu, thus mirroring the Virchow's triad (hypercoagulability, stasis and endothelial injury) (5, 51). Remarkably, there is an imbalance of low concentrations of blood coagulation factors (e.g., factor V and VIII) and high concentrations of anticoagulant molecules (e.g., TFPI, antithrombin) in the lymph. The production and release of tissue plasminogen activator tPA and PAI-1by lymphatic endothelial cells exhibit high fibrinolytic activity. The fibrin generation is largely counteracted by the unavailability of cell surface anionic phospholipids such as those physiologically present on blood platelets, combined with only low levels of coagulation factors, and the strong inhibitory activity of heparin, antithrombin, and tissue factor pathway inhibitor (46). The above mechanisms may be the reasons why lymphatic coagulation is slower than blood coagulation (52, 53).

Theoretically, any known cause of lymphatic vessel occlusion due to internal obliteration, external compression, or increased lymphatic pressure may predispose an individual to localized lymphatic thrombosis (46). The venous pressure is higher than the lymphatic pressure, and this may prevent further reflux of blood into the lymph vessels to protect lymphatic function in cases of lymphovenous valve dysfunction (54, 55). However, lymphatic valves dysfunction may cause human lymphedema or reflux of blood into the terminal parts of lymph ducts, followed by coagulation. A recent experimental study revealed a new pathway for platelet activation induced by the endothelial layer of the lymph vessels. Platelet aggregation stabilizes thrombin to prevent retrograde blood flow, resulting in thrombus formation at the lymphovenous valve (56).

## Coagulation in Different Lymphatic Vessels

Lymphatic vasculature displays remarkable heterogeneity in structure and function. The primarylymphaticsare right lymphatic duct (RLD) and thoracic duct (TD). TD is the largest lymphatic vessel that drains chylous and lymphatic fluid from most of the human body including the limbs and abdomen (57). The liver produces about 25–50% of the lymph flowing through the TD (58). The liver is also the primary site of synthesis of all clotting factors and their inhibitors, as well as several proteins involved in fibrinolysis and anticoagulation (59, 60). This may explain why the TD contains far more proteins and clotting factors than the lymphatic ducts in the limbs, including the axillary or inguinal lymphatics.

The analyses of lymphatic vessels inembryos, an dual origin theory proposed that lymphatic endothelial cells originated from two sources (61, 62). The study of mouse embryos and Xenopus tadpoles also provide evidence that lymphatic endothelial cells origin from both the veins and scattered mesodermal precursor cell (63, 64). These studies demonstrate the existence of a separate population of lymphatic endothelial cells with distinct molecular and function identity that forms local lymphatic vessels (65). Understanding the different lymphatic endothelial cells sources is crucial, as distinct lymphatic endothelial cells will likely contribute differentially to lymphatic thrombosis.

In contrast to blood, which is not in direct contact with the cellular layers of parenchymal organs, the lymph is derived directly from the organ interstitialfluid, whichbathes each organ's cellular layers (66, 67). That is, the lymph can actually provide the different organ's specific metabolic signature. The analysis of the proteome, lipidome, and metabolome of lymph collection from different parenchymal organs demonstrated differences in the distribution of lymphatic fluid from different anatomical areas (1). The composition of coagulation factors in lymph also depends on the interstitial fluid in the surrounding tissues. In addition, hemodynamic forces, pathological conditions, and the extracellular environment may affect the lymphatic system, leading to the adaptive changes (68). This may result in distinct levels and patterns of coagulation of lymphatic vessels in specific organs.

## Different Hemostatic Properties for Lymphatic and Blood Coagulation

### Platelets

It is well-known that the platelets play an essential role in the formation of white thrombus in circulating blood (30). The lymph contains noplatelets yet it is known that thrombosis may occurwithin lymphatics (42, 50, 51). Although platelets are absent from the lymph, the human thoracic duct lymph contains phospholipidcomponents similar to those present at the plateletsurface. It is also suggested that the lymphocytespresent in the lymph were efficient surrogates of bloodplatelets during lymphaticthrombosis (46, 69). However, clearphysiologicalevidence for this theory is still lacking.

In the coagulation cascade of the blood, the embolus is adherent to the vascular walls through platelet, leading to vessel occlusion (39). The interaction between matrix-bound vWF and its platelet receptor, adhesion glycoprotein Ib-IX (GPIb-IX), is required for initial platelet adhesion (70). The lymph does not contain platelets and its adhesionglycoproteinIb-IXcomplex, therefore thrombus may stay in the lumen and not adhere to the lymphatic wall. To date the location of the lymphatic thrombus has not been thoroughly investigated. However, the lymphatic thrombus is usually retained within the regional lymph nodes (46). In contrast, dislodgement of the embolism from the blood vessel may lead to distant vessel occlusive diseases (71).

### Von Willebrand Factor

According to the existing literature, the ultrastructure of lymphatic vessels in healthy humans does not contain WPBs. Von Willebrand antigen was present in a very low concentration in the rabbit limb lymph, primarily as low molecular weight multimers (43). The presence of vWF in human dental pulp lymph has been reported (72, 73), while other studies found no lymphatic vessels in human dental pulp (74). The lymphatic vascular hemophilia factor vWF is most likely to be produced by lymphoendothelial cells at low concentrations, which may also slow the formationof lymphatic thrombosis (37, 75–77). A low concentration of vWF in lymphatic fluid may prevent the development of lymphoid thrombosis (52).

### Coagulation Factors

As early as 1980, coagulation factors in thoracic-duct lymph of dogs were found to have significantly different coagulation activity from that typically measured in plasma (78). Both activity and concentration of most coagulation factors wereconfirmed to be dramatically reduced in lymph as comparedwith plasma (53). The percentages in lymph as compared with plasma were 5–20% activity and 20–40% antigen for factor V, factor VII, factor VIII, factor IX, factor X, fibrinogen andprothrombin (53, 79). The activity of factor VII in limb lymph was lowerthan the activity of factor X and of prothrombin despitethe similar molecular weights and other properties ofthese three proteins (43). In animal experimentson lymph node transplantation in hemophilia dogs, data have revealed that lymph nodes have the ability to produce factor VIII and that lymphatic endothelial cells are the main source of factor VIII in extrahepatic tissue (80, 81). Especially, the lymph fibrinogen level of almost 30% of the mean plasma level (43). However, the fibrin generation issubstantially inhibited by the unavailability of cell surface anionic phospholipids under physiological conditionsin lymph, essentially making the lymph a hypocoagulable biological fluid. Moreover the low levels of factor VIII and factor V prevent the activation of factor X, thus preventing the generation of fibrin in lymph (43).

### D-Dimers

The concentrations of coagulation factors and anticoagulation factors in the lymph were much lower than those in the plasma, but the concentration of d-dimer in the lymph was higher than that in the plasma, often more than fivefold (46, 53). Bach-Gansmo et al. have demonstrated that high d-dimerconcentrationsin the lymph occur during fibrinogen degradation mediated by human neutrophil elastase (82). This high level in lymph was notexploredbut may indicate proteolysis of fibrinogen and fibrin with release of D-like and D-dimerlike fragments in interstitial fluid (53).

### Antithrombins

Lymphaticfluid is thought to have weaker clotting capability thanblood because it contains little thrombokinase and highlevels of antithrombin (42, 53). Althroughboth plasma and lymph contained only very low concentrations of antithrombin-factor Xa complexes, their concentration in lymph exceeded their concentration in plasma. Moreover, anticoagulantlyactive glycosaminoglycans on lymph endothelium thatcould markedly potentiate antithrombin activity also mayplay an important role in preventinglymphatic thrombosis *in vivo* (43).

### TFPIs

The lymph was found to contain two main anticoagulant protease inhibitors, antithrombin and TFPI. TFPI-Xa complex concentrations were higher in lymph than plasma, and mean lymph TFPI antigen in lymph was approximately twice the mean lymph TFPI activity. These results suggested factor X activation in interstitial fluid followed by its inactivation by TFPI. And TFPI-Xa complex inactivates the catalytic activity of VII-TF and prevents extravascular VIII-TF activating factor X from progressing to the generation of fibrin in the interstitial fluid and lymph of peripheral tissues (43, 53). It usually takes longer for clotting to occur in the lymph than in the blood (47) (Table 1).

**Table 1:** Hemostatic propertyin human plasma and lymph.

	**Plasma**	**Lymph**	**References**
Platelets	Yes	No	(42)
von Willebrand factor	High	Low	(52)
Coagulation factors activity	High	Low	(43)
D-dimers activity	Low	High	(53)
Antithrombins activity	Low	High	(79)
TFPIs activity	Low	High	(43)

### Lymphatic Endothelial Cellspecific Molecules

In recent studies, the lymphatic endothelial cellsspecific membrane markers includinglymphatic vessel endothelial hyaluronan receptor 1 (LYVE-1), podoplanin, Vascular endothelial growth factor receptor-3 (VEGFR-3) and specific intracellular factors such as Prox-1 have been found (83–85). The specific factors have been suggested as being able to permit the discrimination between blood and lymphatic microcapillaries inhistological sections (86). However, whether these special molecules may be the inducement of lymphatic thrombosis remains to be clarified. Podoplanin is expressed in lymphatic endothelial cells and its expression is maintained by prosperohomeobox protein 1 (Prox1). Under inflammatory conditions, podoplanin expression is increased in lymphatic endothelial cells. In pathological status, the ectopic podoplanin expression is induced, whereas ectopic podoplanin-expressing cells migrate to the vicinity of vascular endothelial cells and interact with hyperpermeable vascular leaky platelets C-type lectin like receptor 2 (CLEC-2) facilitates thrombus formation (87). This pathologically expressed podoplanin may also participate in lymphatic thrombosis via interaction with leaky platelet CLEC-2, while it needs further investigation. Vascular endothelial growth factor (VEGF) was discovered by the use of a coagulation assay in 1990 as a factor that explains the localization of endothelium-dependent fibrin formation in tumors after TNF treatment (88). The urokinase-type plasminogen activator (u-PA) and PAI are actives by VEGF (89) and tissue factor was demonstrated to control VEGF expression (90). A missense mutation in the tyrosine-kinase domain of the VEGFR-3 ligand cause primary congenital lymphedema (91), leading to prolonged pressure to formation lymphatic obstruction. The production and activation of VEGF and its receptor suggest their involvement in lymphatic thrombosis (92). An as yet unknown cascade of lymphatic thrombosis evoked by the lymphatic specific factor VEGFR-3 either alone or in conjunction with other control elements awaits further exploration.

## Lymphatic Thrombosis-Related Diseases

### Lymphedema

Lymphedema is a chronic and persistent disease that can easily lead to a large number of comorbidities (93). It may be caused by mechanical obstruction or destruction of the lymphatic wall that leads to abnormal accumulation and overloading of interstitial fluid containing high-molecular-weight proteins (94). Lymphedema is the long-term stasis of fluid in the lymphatic vessels, causing lymph nodes to continue to contract due to the increase in volume. In lymphedema, lymphatic valve function and smooth muscle contraction gradually deteriorate, leading to the weakening of unilateral lymph fluid propulsion, forming a vicious circle; the continuous swelling in the lymphatic vessels and the blocking of proteins lead to fibrosis, which might trigger the formation of fibrinoid and occlusive thrombosis in the lumen (46, 95). In severe cases, cellulitis may occur (96). However, lymphatic injury and dissection are not the only causes of lymphedema (97). Some studies have shown that mutations in coagulation factor V combined with other susceptible factors may cause lymphatic thrombosis and lead to lymphedema (98). The causal association between thrombus and lymphedema is unclear. One possibility is that lymphatic thrombosis develops first and induces lymphedema. Another possibility is that lymphatic stasis and lymphedema develop first, and thrombus forms due to reduced low flow in the occluded lymphatic vessels (50). Nevertheless, it is certain that the edema is caused, at least in part, by interruption of lymphatic flow (42, 49).

### Amyloidosis

In cases studies of amyloidosis, a total of 2% of patients diagnosed with all types of amyloidosis had lymph node amyloidosis (99, 100). Both von Willebrand factor and factor V were identifiable in areas of lymph node amyloid deposition by immunohistochemistry. This research providing evidence that the adsorption of coagulation factors from the circulation into the lymphatic vessels by extracellular deposition of pathologic amyloid. This pathological changes result in an acquired factor deficiency and thus the cause of the bleeding disorder (101). The adsorption of coagulation factors from the circulation into the lymph by amyloid lymphadenopathy may lead to the formation of lymphatic thrombosis. These studies may help explain the unclear pathogenesis of lymphadenopathy and related lymphatic thrombosis.

### Infection

In an experiment on liver and gallbladder edema caused by cantharidin, liver lymphatic vessels expanded extensively. Lymphatic endothelial cells are damaged and denatured under the action of poison, which forms a fibrous network in lymphatic vessels to block lymph flow (82). Other fungal, bacterial and viral infectionsmay cause cellulitis or progressive lymphatic destruction, which sporadically predispose to thrombosis of lymph vessels. The common infections are lymphatic filariasis or sustained by chlamydia trachomatis, mycobacterium tuberculosis, treponemapallidum, or streptococcus pyogenes (46). Among them, the inflammatory response and lymphatic endothelial damage triggered by the nematode infection is a major factor in the pathogenesis of lymphaticthrombosis in lymphatic filariasis (102, 103).

### Cancer

Different mechanisms may lead to lymphatic thrombosis or occlusion in cancer patients, including external compression from tumor masses, neoplastic occlusion of lymphatic vessels by metastatic cells or lymphatic dysfunction afterlymphadenectomy (46). Coagulation after lymph node dissection continues to be a frequently reported morbidity (104). The tissue injury from surgery releases tissue factor that may cause hypercoagulability in the surrounding lymph. The outflow obstruction induced by removal of axillary lymphatics draining the arm would be generated stasis of lymphovenous channels. The pathology demonstrated fibrin clot in lymphatics of biopsied axillary webs (105). Therefore, lymphatic thrombosis maybe a significant cause of axillary web syndrome in the early postoperative period for patients after axillary lymph node dissection.

### Sporadiccases

Patients with chronic venous insufficiency, deep vein thrombosis, venous valve damage may develop persistent inflammation and chronic damage to the lymphatic vessel, leading to loss of lymphatic vessel contraction function, reduced lymphatic drainage, and severe lymphatic thrombosis (106). The condition is often complicated by concurrent cellulitis and inflammation of the distal lower limb (95). When the function of the lymphovenous valve is impaired, platelet-mediated thrombosis occurs, preventing further lymphatic venous reflux, producing lymphatic stasis, and causing lymphatic obstruction (107). However, this may lead to thoracic outlet syndrome or complications that require central venous catheterization or coronary artery bypass grafting (108, 109). Congenital or acquired thoracic duct outflow obstruction (secondary to central venous thrombosis or injury during cardiothoracic surgery) is the cause of high morbidity and mortality in newborns (110).

## Discussion

Although lymphatic thrombosis occurs in a variety of diseases, it has rarely been reported. For cancer lymphatic thrombosis, the leading causes include external tumor compression, neoplastic obliteration by metastatic cells, or lymphatic injury after lymph node dissection. Patients with fungal, bacterial and viral infections in tissues near lymphatic vessels may have lymphatic thrombosis and progressive lymphatic destruction. Lymphatic thrombosis was also found in sporadic cases of complications of central venous thrombosis or injury during cardiothoracic surgery, chronic venous insufficiency and thoracic outlet syndrome, which accompanied with lymphatic venous valve injury or obstruction of thoracic catheter drainage. If the lymphatics thrombosis causes accumulation of interstitial fluid, the lymphatico-venous anastomosis is expected to reduce the inner pressure of the lymphatic vessel and reconstruct lymph flow. For patients with severe, unexplained edema, clinicians may consider the possibility of the lymphatic thrombosis. Indocyanine green lymphography and radionuclide lymphoscintigraphy may locate lymphatic obstruction, while laboratory tests are still limited. Many cases of lymphatics thrombosis were found in lymphatic biopsy. Further studies are needed to understand the mechanisms of lymphatic thrombosis and develop novel diagnostic and therapeutic strategies.

## Author Contributions

JLiu conceived and designed the review. JLiu, WZ, and JT wrote the first draft. JLi, JLia, and XQ participated in writing of the manuscript. All authors contributed to the article and approved the submitted version.

## Funding

This study was supported by the National Nature Science Foundation of China (81873473 and 91939110), Academic Promotion Program of Shandong First Medical University (2019QL014) and Shandong Taishan Scholarship (JLiu).

## Conflict of Interest

The authors declare that the research was conducted in the absence of any commercial or financial relationships that could be construed as a potential conflict of interest.

## Publisher's Note

All claims expressed in this article are solely those of the authors and do not necessarily represent those of their affiliated organizations, or those of the publisher, the editors and the reviewers. Any product that may be evaluated in this article, or claim that may be made by its manufacturer, is not guaranteed or endorsed by the publisher.

## Abbreviations

CLEC-2: C-type lectin like receptor 2
GPIb-IX: GlycoproteinIb-Ix
LYVE-1: Lymphatic vessel endothelial hyaluronan receptor 1
PAI: Plasminogen activator inhibitor
Prox1: Prospero homeobox protein 1
RLD: The right lymphatic duct
TD: The thoracic duct
TF: Tissue factor
TFPI: Tissue factor pathway inhibitor
TM: Thromboregulatory protein
tPA: Tissue plasminogen activator
u-PA: Urokinase-type plasminogen activator
VEGF: Vascular endothelial growth factor
VEGFR-3: Vascular endothelial growth factor receptor 3
vWF: Von willebrand factor
WPBs: Weibel-palade bodies.
